# Regulation of human glioma cell migration, tumor growth, and stemness gene expression using a Lck targeted inhibitor

**DOI:** 10.1038/s41388-018-0546-z

**Published:** 2018-10-23

**Authors:** J. P. Zepecki, K. M. Snyder, M. M. Moreno, E. Fajardo, A. Fiser, J. Ness, A. Sarkar, S. A. Toms, N. Tapinos

**Affiliations:** 1 Molecular Neuroscience & Neuro-Oncology Laboratory, Brown University, Providence, RI USA; 2 University of Minnesota, College of Veterinary Medicine, St. Paul, MN USA; 3 Department of Systems and Computational Biology, Albert Einstein College of Medicine, Bronx, NY USA; 4 Department of Biological Sciences, Messiah College, Mechanicsburg, PA USA; 5 Department of Neurosurgery, Geisinger Clinic, Danville, PA USA; 6 Department of Neurosurgery, Brown University, Rhode Island Hospital, Providence, RI USA

**Keywords:** CNS cancer, Cancer stem cells, Mechanisms of disease

## Abstract

Migration of human glioma cells (hGCs) within the brain parenchyma makes glioblastoma one of the most aggressive and lethal tumors. Studies of the cellular and molecular mechanisms underlying hGC migration are hindered by the limitations of existing glioma models. Here we developed a dorsal root ganglion axon-oligodendrocyte-hGC co-culture to study in real time the migration and interaction of hGCs with their microenvironment. hGCs interact with myelinated and non-myelinated axons through the formation of pseudopodia. Isolation of pseudopodia-localized polysome-bound RNA reveals transcripts of *Lck, Paxillin, Crk-II*, and *Rac1* that undergo local translation. Inhibition of Lck phosphorylation using a small-molecule inhibitor (Lck-I), blocks the phosphorylation of Paxillin and Crk-II, the formation of pseudopodia and the migration of hGCs. In vivo intraventricular administration of the Lck-I using an orthotopic xenograft glioma model, results in statistically significant inhibition of tumor size and significant down-regulation of Nanog-targeted genes, which are associated with glioblastoma patient survival. Moreover, treatment of human glioma stem cells (hGSCs) with Lck-I, results in significant inhibition of self-renewal and tumor-sphere formation. The involvement of Lck in different levels of glioma malignant progression, such as migration, tumor growth, and regulation of cancer stemness, makes Lck a potentially important therapeutic target for human glioblastomas.

## Introduction

Human glioblastoma is one of the most aggressive and lethal cancers due to the presence of tumor-propagating human glioma stem cells (hGSCs) and the highly migratory nature of these cells [1, 2]. Recent studies have revealed extensive intratumoral heterogeneity in transcript expression, which likely contributes to treatment failure and tumor recurrence [3, 4]. Despite advances in understanding the basic biology of glioblastoma, studies on glioma cell migration are hindered by the lack of efficient in vitro or in vivo migration models. Until now studies on human glioma cell (hGC) migration were performed on Laminin coated surfaces [5], collagen and astrocyte layers [6], extracellular matrix layers [7], electro spun nanofibers [8], or postmortem in mouse xenograft models [7]. Although all these studies have provided information on the migratory properties of hGCs, their real-time interaction with myelinated and non-myelinated axons has not been studied. Migration of hGCs occurs in the brain parenchyma through continuous interaction with axon fibers, glial cells, microglia, and endothelial cells, which likely affect their migratory efficiency and cannot be accounted for with the current monolayer migration models.

Here, we present a novel approach to study hGC migration on myelinated and non-myelinated axons. Building upon our experience with dorsal root ganglia (DRG) axon Schwann cell co-cultures [9], we developed an ex vivo system containing DRG axon-oligodendrocyte co-cultures and hGCs. We show in real time that hGCs interact with axonal tracks and migrate along the myelinated and non-myelinated axons. In addition, we observed that hGCs interact with neighboring axons through extensive formation of pseudopodia. Using a previously described Boyden chamber system [10] we isolated the hGC pseudopodia and performed polyribosome fractionation followed by qPCR and immunoblotting to detect transcripts that are being translated locally and could regulate pseudopodia formation and the interaction of hGCs with axons. We discovered local translation of Lck, Paxillin, Crk-II, and Rac1. Next, using the TCGA database we showed that Lck mRNA is overexpressed in Grade IV tumors and in tumors with wild-type IDH. Inhibition of Lck activity blocks phosphorylation of paxillin, Crk-II, the formation of pseudopodia and the in vitro migration of hGCs. Moreover, in vivo intraventricular delivery of a small molecule inhibitor of Lck (Lck-I) using an orthotopic xenograft mouse model results in significant reduction of tumor size. RNA sequencing of microdissected xenografted tumors revealed that sustained local treatment with Lck-I results in significant inhibition of Nanog-targeted genes, which are associated with decreased patient survival rates [11, 12]. Finally, in vitro treatment of hGSCs with Lck-I results in significant inhibition of self-renewal and tumor-sphere formation. Based on our data, we propose that Lck is a therapeutic target for human glioblastomas.

## Results

### Development of ex vivo co-culture system to study interaction of hGCs with axons

hGSCs were isolated from glioblastoma surgical resection as described previously [13]. The hGSCs were cultured as floating neurospheres and subjected to limiting dilution and self-renewal to generate, secondary and tertiary neurospheres. hGSCs were stained positive for the stem cell markers nestin, CD133, Musashi 1, Nanog, and Sox 2 (Supplementary Figure S1A). hGSCs are capable of differentiating to GFAP+ astrocytes, A2B5+ oligodendrocytes and NeuN+ cells in vitro (Supplementary Figure S1B). To examine if the Cd133+/Nestin+ hGSCs can recapitulate the complexity and heterogeneity of the original tumor they were transplanted into the brain of immunocompromised mice under stereotactic guidance. Four weeks after transplantation mice were euthanized and tumors were stained with H&E or the proliferative marker Ki67, the GFAP astrocytic marker and the Olig2 oligodendrocyte marker (Supplementary Figure S1C). This showed that our hGSCs maintain the ability to generate heterogenic tumors in animals.

**Fig. 1:**
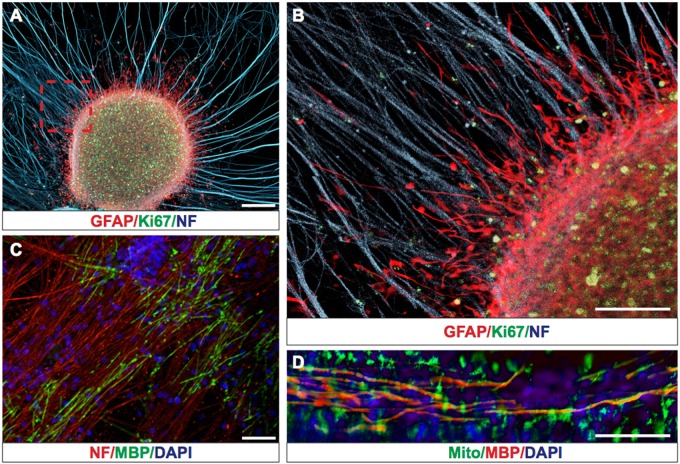
hGCs form tumor-like structures and migrate along non-myelinated and myelinated axonal tracks on an ex vivo co-culture system. **a** Representative picture of hGCs forming GFAP^+(red)^/Ki67^+(green)^ tumor-like structures on DRG axons expressing Neurofilament (NF blue). **b** Magnified area from **a** (red rectangle) showing GFAP^+(red)^ human glioma cells interacting and migrating along non-myelinating axons NF^+(blue)^. **c** Establishment of myelinated MBP^+(green)^ axonal tracks after addition of oligodendrocyte progenitor cells on purified DRG axons (stained red with Neurofilament antibody). **d** Representative picture of hGCs stained with human Mitochondrial marker (Mito^+(green)^) migrating along myelinated axonal tracks (MBP^+(red)^ and nuclei counterstained with DAPI^(blue)^). Scale bars: 200 μm

To study the interaction of hGCs with axons we generated purified DRG axons as previously described [14, 15]. Next, we seeded the purified DRG axons with hGCs, which exhibited extensive interactions with the unmyelinated DRG axons, integrated within the axonal network and formed GFAP+/Ki67+ tumor-like structures (Fig. 1a, b). To determine how hGCs interact with myelinated axons, we seeded DRG axon cultures with purified rat oligodendrocytes and induced myelination as previously described [16, 17]. The DRG axon oligodendrocyte co-cultures were stained with myelin basic protein (MBP) antibodies to detect compact myelin (Fig. 1c). Addition of hGCs on the myelinated DRG-oligodendrocyte co-cultures showed that hGCs migrate in association with myelinated axons (Fig. 1d).

### hGCs interact with axons through the formation of pseudopodia

**Fig. 2:**
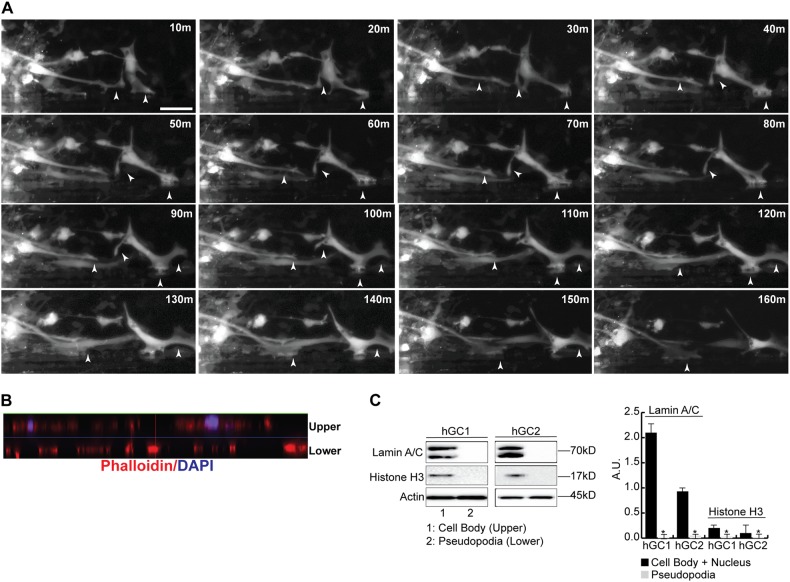
Human glioma cells interact with axons through the formation of pseudopodia. **a** hGCs were seeded on purified DRG axons and live cell imaging was performed with images acquired every 10 min using a Zeiss Axiovert microscope equipped with the AxioVision Software. Dynamic extension and retraction of pseudopodia were seen (arrowheads) as the hGCs migrated along the axons. **b** Side-view of 10-slice z-stack image of the Boyden chamber (30 μm depth) shows that only the phalloidin^+(red)^ hGC pseudopodia cross the 1 μm pore (lower) while the DAPI^+^ nuclei and the rest of the cell body remain within the upper chamber (upper). The thin blue line (pseudocolored) in the middle of the z-stack represents the membrane separating the upper from the lower chambers. **c** Representative Western blots using proteins isolated from the upper and lower chambers show that the pseudopodia fraction (lower chamber) is pure and does not express the nuclear markers Lamin A/C and Histone H3 that are present in nuclei isolated from the upper chamber. The experiments were repeated three times and significance was calculated with a Student’s *t-*test (*p < 0.05)

To study the interaction of hGCs with non-myelinated and myelinated axons in real time, we infected hGCs with a Lentivirus expressing GFP, seeded the Lenti-GFP-hGCs on purified DRG axons and performed live imaging for 5 days. hGCs interact with and invade the axonal network forming extensive pseudopodia that associate with axons sometimes “pulling” and deforming individual axon fibers with their pseudopodia (Supplementary Movie S1). Next, we prepared DRG-oligodendrocyte myelinated axonal tracks in parallel orientation using Campenot chambers on a collagen substrate with parallel scratches for guidance [16]. Time-lapse photography of hGCs on parallel myelinated axons shows the active formation and retraction of multiple pseudopodia as the hGCs interact with the myelinated fibers (Fig. 2a). hGC pseudopodia were isolated using a Boyden chamber separated by a membrane with 1 μm pore openings [10, 18]. Confocal z-stack images of the chambers show that only the hGC pseudopodia cross the 1 μm pore while the nuclei and the rest of the cell body remain within the upper chamber (Fig. 2b). Protein isolation from the upper and lower chambers shows that the pseudopodia fraction is pure and does not express the nuclear markers Lamin A/C and Histone H3 (Fig. 2c).

### hGC pseudopodia contain migration-specific RNA transcripts

**Fig. 3:**
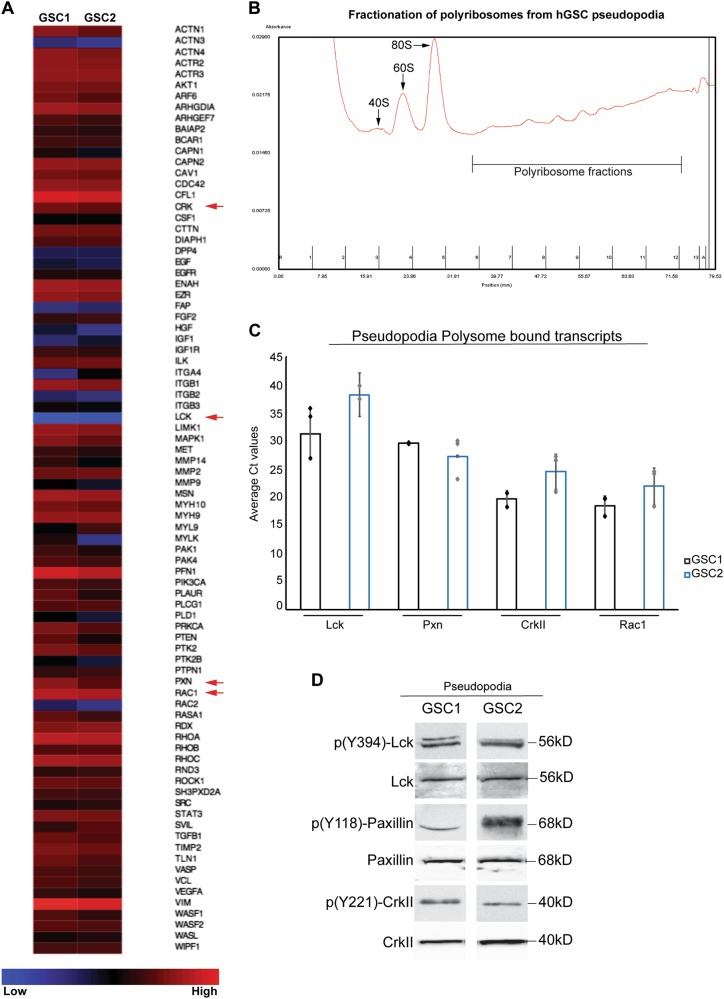
Localized expression and translation of Lck-regulated migration transcripts in hGC pseudopodia. **a** RNA from purified pseudopodia of hGCs from two patients (GSC1) and (GSC2) with glioblastomas was used to perform a qPCR array for migration-specific genes (SA Biosciences). Heat map expression analysis shows that RNA isolated from pseudopodia of hGCs contains various migration-specific transcripts including Lck, Paxillin, CrkII, and Rac1 (arrows). **b** Polysome profile of hGC pseudopodia following sucrose density gradient fractionation. The presence of 40S, 60S, 80S, and polyribosomes is noted on the profile. **c** Cycle threshold (Ct) values of Lck, Paxillin, CrkII, and Rac1 transcripts bound to polyribosomes in hGC pseudopodia. The Ct values were obtained from three independent experiments for each hGC sample. The graph shows the individual data points for each experiment and the bars represent the mean Ct values ± standard deviation. **d** Western blotting for the detection of p(Y394)-Lck, p(Y118)-Paxillin, p(Y221)-CrkII, and the corresponding total proteins as loading controls in protein lysates isolated from pseudopodia of two patients derived hGCs, shows the local presence of the phosphorylated proteins. The experiment was repeated three times for each hGC

We isolated RNA from the purified pseudopodia and performed a qPCR array for migration-specific genes (SA Biosciences). We used RNA from hGCs of two patients, representing different subtypes of glioblastomas according to the TCGA classification [19] and their mutational profile (Supplementary Table 3). We demonstrate that RNA isolated from pseudopodia of hGCs contains migration-specific transcripts (Fig. 3a) including Cdc42, Cofilin-1, Ezrin, Ilk, Limk, STAT3, MMP2, and Itgb1, recently described as part of intracellular pathways related to glioblastoma migration [20–24]. In addition, we identified expression of Lck, Paxillin, CrkII, and Rac1 transcripts in the hGC pseudopodia (Fig. 3a), which we have recently shown to regulate migration of Schwann cells in the peripheral nervous system [25]. Migration-specific transcripts including Lck, Paxillin, CrkII, and Rac1 were also highly expressed in RNA-seq data from hGSCs from three patients with different subtypes of glioblastoma (Patients 1–3), as compared with transcript expression of human neural stem cells (HNSCs) derived from H9 embryonic stem cells (Supplementary Figure S2A).

### Transcripts of lck, paxillin, crkii, and Rac1 associate with local polyribosomes in pseudopodia of hGCs

We isolated polyribosomes using sucrose density gradient fractionation from hGC pseudopodia [26] (Fig. 3b), followed by RNA isolation from the isolated polyribosome fractions. Using qPCR, we detected the presence of Lck, Paxillin, CrkII, and Rac1 transcripts bound to the local polyribosomes in hGC pseudopodia (Fig. 3c), suggesting that these transcripts may undergo local translation. Finally, we performed protein isolations from purified hGC pseudopodia and detected the expression of phosphorylated Lck, Paxillin, and CrkII within the pseudopodia (Fig. 3d) suggesting a local functional role of these kinases in hGCs.

### Lck is highly expressed in human glioblastomas

**Fig. 4:**
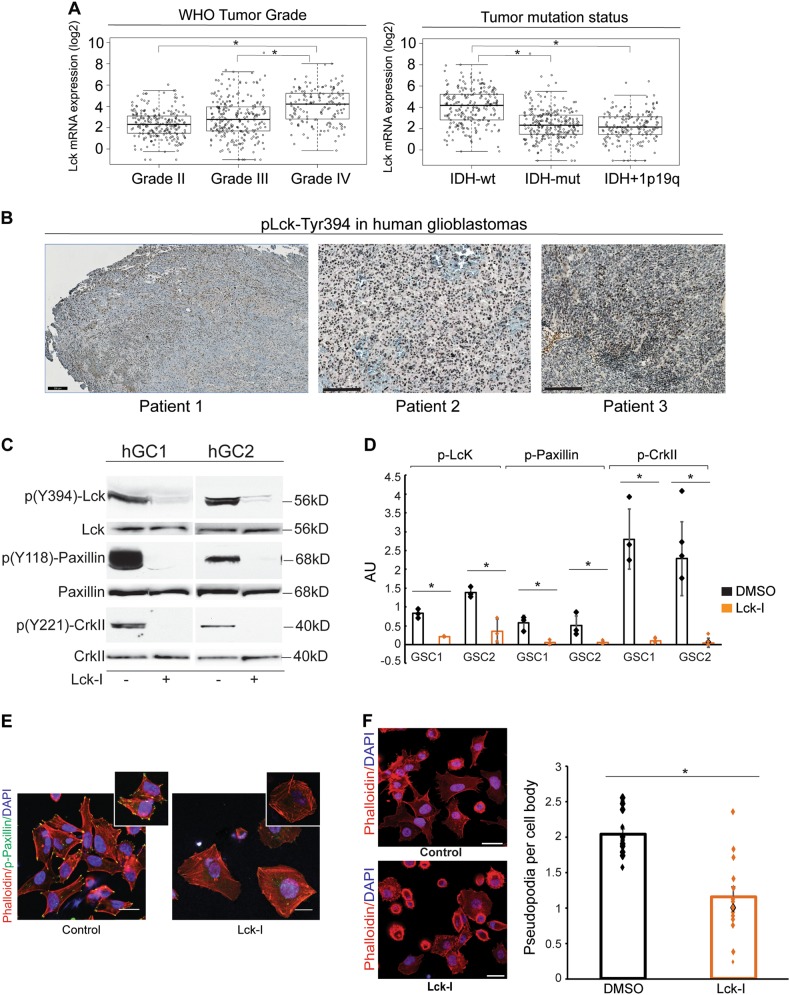
Phospo-Lck is expressed in human glioblastomas and inhibition of phospho-Lck results in inhibition of paxillin and CrkII activation and loss of pseudopodia formation in hGCs. **a** Analysis of TCGA database shows that Lck mRNA is significantly upregulated in glioblastomas compared to Grade II and III tumors. In addition, the expression of Lck mRNA is significantly higher in glioblastomas with wild type IDH as compared to mutated IDH or tumors with mutated IDH and 1p/19q co-deletion (*p < 0.0001, One-way Anova). **b** Immunohistochemistry using pLck-Tyr394 antibody shows widespread expression of pLck in human glioblastoma tissue sections. **c** and **d** hGCs isolated from two patients with glioblastomas (hGC1 and hGC2) were left untreated or treated with 500 nM Lck-I for 2 h. Lysates were analyzed for phospho(Y394)-Lck, phospho(Y118)-paxillin, phospho(Y221)-CrkII, and the corresponding non-phosphorylated proteins as loading controls. Treatment with Lck-I significantly reduced the phosphorylation levels of Lck, paxillin, and CrkII (graph shows individual densitometric measurements from three experiments, the bars represent mean values ± s.d., *p < 0.05 with two-tailed Student’s *t*-test). **e** hGCs were stained for phalloidin-rhodamine and phospho(Y118)-paxillin (green) to identify pseudopodia and active paxillin. Paxillin phosphorylation was reduced in Lck inhibitor-treated cultures from the tips of hGC pseudopodia as compared to control cultures. Insets show individual control cells with positive *p*-Paxillin at the tips of pseudopodia while in Lck-I-treated cells we did not observe *p*-Paxillin or the formation of pseudopodia. **f** Numbers of pseudopodia per cell were counted (30 cells per well, individual measurements from 12 independent wells are plotted, bars represent mean values ± s.d.). Treatment with Lck inhibitor significantly reduced the number of pseudopodia per cell (*p* < 0.05, calculated with two-tailed Student’s *t*-test). Scale bars represent 200 μm

To determine the expression of Lck in human glioblastomas we searched the TCGA database (http://gliovis.bioinfo.cnio.es/) and plotted the expression of Lck mRNA segregated according to WHO tumor grade and IDH/1p19q status. This showed that Lck mRNA is significantly upregulated in glioblastomas compared to Grade II and III tumors. In addition, the expression of Lck mRNA is significantly higher in glioblastomas with wild type IDH as compared to mutated IDH or tumors with mutated IDH and 1p/19q co-deletion (Fig. 4a). Finally, Lck transcript expression is significantly higher in glioblastomas compared to normal brain tissue (Supplementary Figure S2B).

Lck is regulated by phosphorylation on multiple residues, including Ser-158 in the SH2 domain [27] and Tyr-394 [28]. We stained human glioblastoma tissue sections with pLck-Tyr394 antibody, which showed extensive staining of hGCs throughout the tumor (Fig. 4b). In addition, glioma tissue arrays containing 40 glioblastoma samples in duplicate (US Biomax, Inc.) were stained with antibody against pLck-Ser158 (Abcam), which showed that 90% of human glioblastomas on the array stained positive (Supplementary Figure S2C).

### Inhibition of Lck blocks the activation of Paxillin and CrkII in hGCs

To elucidate the role of phospho-Lck signaling on paxillin and CrkII in hGCs we used an Lck inhibitor (A770041, Axon Medchem) that specifically binds to the Lck-active site at nanomolar concentrations. Lck-I exhibits 8-fold, 60-fold, and 300-fold specificity for Lck over Src kinase family members Lyn, Src, and Fyn, respectively [29]. A kinase interaction map of A770041 was profiled through the KINOMEscan project, which showed that A770041 can bind with high affinity 35 kinases when used at 1 µM concentration [30]. However, A770041 shows greater than 200-fold selectivity against a battery of ~20 serine/threonine and tyrosine kinases outside of the Src family and IC_50_ values greater than 10 µM in a CEREP panel of ~70 molecular targets [29]. Based on these data, the maximum concentration used in our studies was 500 nM, which showed no effect on the phosphorylation state of Src, Yes, Lyn, and Fyn in hGCs (Supplementary Figure S2D). hGCs treated with 500 nM Lck-I show a significant reduction in total levels of phospho(Y394)-Lck, phospho(Y118)-paxillin, and phospho(Y221)-CrkII (Fig. 4c, d, *p* < 0.005). Paxillin is a primary molecular adaptor protein, which localizes to focal adhesion contacts, and stimulates pseudopodia formation and cytoskeletal rearrangement [31]. Treatment of hGCs with the Lck-I results in almost complete absence of phospho-paxillin(Y118) from focal adhesions and pseudopodia (Fig. 4e).

### Lck-I regulates the formation of pseudopodia and the migration of hGCs

**Fig. 5:**
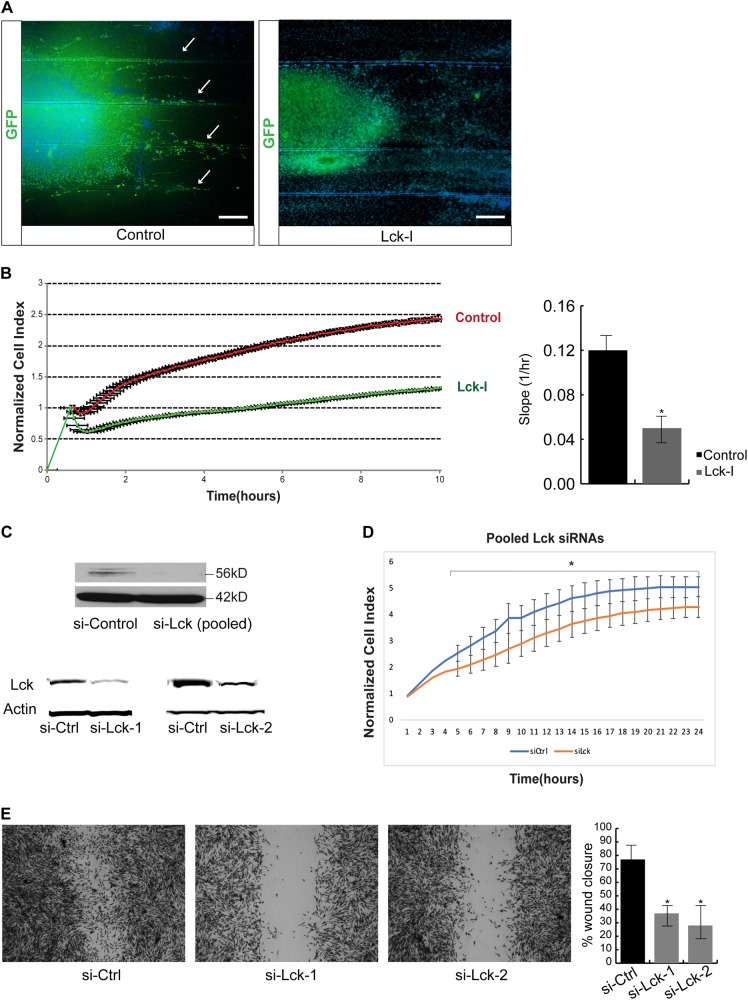
Inhibition of phospho-Lck results in significant reduction of human glioma cell migration. **a** Lenti-GFP infected hGCs were seeded on DRG axons grown on collagen-coated glass coverslips with parallel scratches. Control hGCs migrate along the parallel axonal bundles (arrows). Treatment of the hGC-DRG axon co-cultures with Lck-I for 72 h results in complete inhibition of migration of human glioma cells along the axonal bundles. **b** hGCs were seeded onto a fibronectin-coated xCELLigence E-plate and allowed to attach for 2 h. Cell spreading was monitored every 15 s following the addition of DMSO (CTL) or 500 nM Lck-I. Addition of Lck-I induced an immediate retraction of cell processes as compared with DMSO-treated cultures and overall the cell index, which corresponds to migrating cells remained significantly decreased after 10 h (*n* = 3, *p < 0.005 by two-tailed Student’s *t*-test). Quantification of the protrusion slope indicating the steepness of the pseudopodia protrusion phase in Lck-I treated and control cells, showed that inhibition of Lck results in significant inhibition of pseudopodia protrusion (*p* < 0.005, *n* = 6, two-tailed Student’s *t*-test). **c** Representative Western blots showing reduced expression of Lck in hGCs following transfection with either a pool of four Lck-specific siRNAs or with two independent Lck targeting siRNAs. Control cells were transfected with non-targeting siRNAs. **d** si-Lck and si-Control transfected hGCs were seeded onto a fibronectin-coated xCELLigence E-plate and allowed to attach for 2 h. Cell spreading was monitored every 15 s for 24 h. Knockdown of Lck induces a significant reduction in cell index, which corresponds to migrating cells as compared to control transfected hGCs (*n* = 3, *p < 0.05 by repeated measures Anova). **e** Wound healing assay using cultures of confluent hGSCs transfected with two independent siRNAs against Lck or with a non-targeting siRNA (control). 24 h post injury the wound is not as efficiently healed in the presence of si-Lck as compared to control cells. The area of the wound was measured at T0 (immediately after the wound) and at T24 (24 h later) using ImageJ64 software and plotted as percentage of the total area at ×10 magnification. The results are the average of four independent experiments and show that 24 h after the wound the control cells migrate and cover a significant portion of the wound area, while the si-Lck -transfected cells do not migrate as efficiently (*p* < 0.01)

The number of pseudopodia formed by Lck-I-treated cells was significantly lower than in cells treated with the dimethylsulfoxide (DMSO) control (Fig. 4f, *p* < 0.005). As inhibition of Lck signaling has a dramatic effect on pseudopodia extension and retraction, we investigated whether Lck may also function to regulate the migration of hGCs. DRG axons grown in parallel as shown before, were seeded with Lenti-GFP^+^ hGCs and treated with Lck-I or DMSO for 72 h. This showed that treatment with Lck-I results in complete absence of migrating glioma cells that is prominent in control cultures (Fig. 5a arrows). To obtain quantitative results of the effect of Lck-I on hGC migration we used the xCELLigence System (ACEA Biosciences) that utilizes a microelectronic biosensor technology to measure the total surface area covered by the cell membrane through detection of electrical impedance [32]. In addition, we performed a wound healing assay using hGCs in the absence or presence of Lck-I. Both assays showed that Lck-I induces statistically significant reduction of hGC migration rate (Fig. 5b and Supplementary Figure S3A) and significant reduction of pseudopodia protrusion quantified by the cell protrusion slope (Fig. 5b, *p* < 0.005). To demonstrate the effect of Lck-I in hGC invasion, we seeded hGCs on 3D Alvetex scaffolds (Reinnervate) and quantified the extent of invasion with or without the addition of Lck-I for 72 h. This showed that Lck-I induces significant inhibition of hGC invasion (Supplementary Figure S3B). Next, we examined if Lck-I affects the survival or proliferation of hGCs. We treated hGCs with Lck-I and measured the percentage of viable cells (cytotoxicity) and the percentage of actively proliferating cells (WST-1 assay) 1 day and 5 days after continuous treatment with Lck-I, which showed that Lck-I has no effect on cytotoxicity or cell proliferation of hGCs (Supplementary Figure S3C & D). Finally, to demonstrate specificity of the Lck-I, we performed transfection of hGCs with Lck siRNAs or control non-targeting siRNAs. This showed efficient knockdown of Lck expression in hGCs using pooled siRNAs against Lck or individual siRNAs (Fig. 5c). In addition, the si-Lck-transfected hGCs exhibited significant reduction in the rate of migration compared to si-control-transfected hGCs (Fig. 5d, e), which suggests that Lck is a specific target that regulates glioma cell migration.

### In vivo administration of Lck-I in an orthotopic xenograft mouse model of human glioblastoma results in significant inhibition of tumor formation

**Fig. 6:**
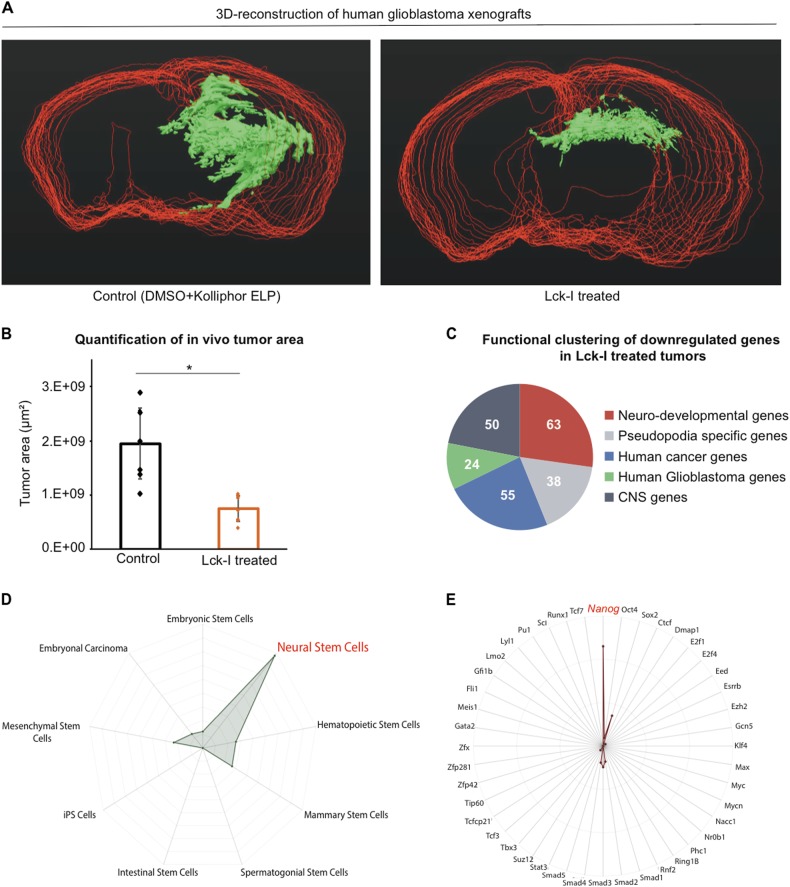
In vivo administration of Lck-I using an orthotopic xenograft model results in significant inhibition of tumor growth and downregulation of stemness gene expression. **a** 3D reconstruction of human glioblastoma xenograft tumors untreated (left panel) or treated with continuous local infusion of Lck-I (right panel) for 4 weeks. Red contours show the outline of the mouse brain and green shows the tumor area as reconstructed using Neurolucida software. **b** Comparison of the total tumor area between Lck-I-treated animals and controls. The results are plotted as mean values ± s.d. (*p < 0.0002, *n* = 16 animals, eight control and eight treated with Lck-I, calculated by two-tailed Student’s *t*-test). **c** Pie chart showing the number of downregulated genes in Lck-I-treated tumors that belong to five functional clusters: neuro-developmental genes, pseudopodia-specific genes, human cancer genes, human glioblastoma genes and CNS genes. Functional annotation was performed using the Broad Institute’s Gene Set Enrichment Analysis tool. **d** Radar chart displaying the overlap between the 743 downregulated genes in tumors treated with Lck-I and the various stem cell types in StemChecker. Significant overlap was observed with stemness signature genes expressed in neural stem cells (red) (*p* < 1.8 × 10^–12^ calculated by the hypergeometric test and adjusted by the Bonferroni correction). **e** Radar chart displaying the overlap between the 743 downregulated genes in tumors treated with Lck-I and genes that are targeted by transcription factors linked to pluripotency and stem cell maintenance (StemChecker). This analysis showed significant overlap of the Lck-I-downregulated genes with Nanog-targeted genes (red) (*p* < 3.5 × 10^–4^ calculated by the hypergeometric test and adjusted by the Bonferroni correction)

To determine the effects of Lck-I on human glioblastomas in vivo, we orthotopically injected 200,000 hGSCs under stereotactic guidance into immunocompromised mouse hosts [33]. The Lck-I was administered through an Alzet pump in the right lateral ventricle continuously for 4 weeks. Control animals received DMSO+ Kolliphor ELP, which is the diluent for the inhibitor. Following the 4-week treatment period, we stained serial brain sections from eight mice treated with Lck-I and eight control animals using HuNu antibody (Abcam) that specifically detects human cells (Neuroscience Associates). In addition, we performed H&E staining to demonstrate the formation of tumors in control and Lck-I treated animals (Supplementary Figure S4). To quantify the effect of the Lck-I on tumor growth, we first stacked and aligned all stained sections from each mouse, we manually traced contours around the HuNu+ cells on each section and combined all sections from each brain to 3D reconstruct the tumors (Fig. 6a and Movies S2 and S3). Analysis of the area of the treated vs. untreated contours showed that treatment with Lck-I results in highly significant reduction in tumor area (Fig. 6b, *n* = 16 animals, *p* < 0.0002 calculated with two-tailed Student’s *t*-test) compared to control animals.

### In vivo treatment with Lck-I results in significant downregulation of cancer stemness gene expression

The extent of in vivo tumor reduction following treatment with Lck-I suggests that the treatment may affect propagation and/or maintenance of hGSCs in addition to its effect on migration. To determine the effect of Lck-I on hGSC transcript expression we treated orthotopically xenografted human glioblastomas in mice with continuous local administration of Lck-I for 4 weeks as described above. At the end of the treatment period, we microdissected the tumors from control animals (*n* = 3, treated with DMSO+ Kolliphor ELP) and Lck-I-treated animals (*n* = 3). RNA isolated from the microdissected tumors was used to perform RNA-seq on Illumina HiSeq2500 (Genewiz). We analyzed ~12,000 expressed genes in our samples and using an adjusted *p*-value < 0.05, we found 744 differentially regulated genes in the animals treated with the Lck-I compared to the controls. The data from this analysis have been deposited in NCBI’s Gene Expression Omnibus [34, 35] and are accessible through GEO Series accession number GSE95289:

To perform functional clustering of the genes in Lck-I-treated tumors we used the Broad Institute’s Gene Set Enrichment Analysis tool:

(http://software.broadinstitute.org/gsea/msigdb/annotate.jsp). The analysis showed that certain subsets of genes in Lck-I-treated tumors exhibit significant overlap with gene sets relevant to neural development, pseudopodia, cancer, glioblastoma, and CNS genes (Fig. 6c). To determine if the Lck-I-modulated genes regulate stemness signatures of hGCs, we used the StemChecker web server. StemChecker compares the uploaded list of genes with those in curated stemness signatures and evaluates the statistical significance of the overlap [36]. We show that the downregulated genes in Lck-I -treated tumors exhibit significant overlap with Neural Stem Cell signature (*p* < 1.8 × 10^–12^) (Fig. 6d). In addition, we examined if the Lck-I-downregulated genes are targeted by transcription factors linked to pluripotency and stem cell maintenance. This showed that the genes inhibited by Lck-I in xenografted human glioblastomas overlap significantly with genes that are targeted by Nanog (*p* < 3.5 × 10^–4^) (Fig. 6e and Supplementary Table 1). Querying the Lck-I inhibited/Nanog-targeted gene dataset to identify genes that belong to the 10% of overexpressed genes in human glioblastomas according to the TCGA, identified 22 transcripts (Supplementary Table 2). To verify the Lck-I-mediated inhibition of these 22 Nanog-targeted transcripts, we performed qPCR 1 day and 5 days after in vitro treatment of hGSCs with Lck-I. This showed, that Lck-I downregulates the expression of most of these transcripts in hGSCs (Supplementary Figure 5).

### Treatment of hGSCs with Lck-I impairs hGSC self-renewal and tumor-sphere formation

**Fig. 7:**
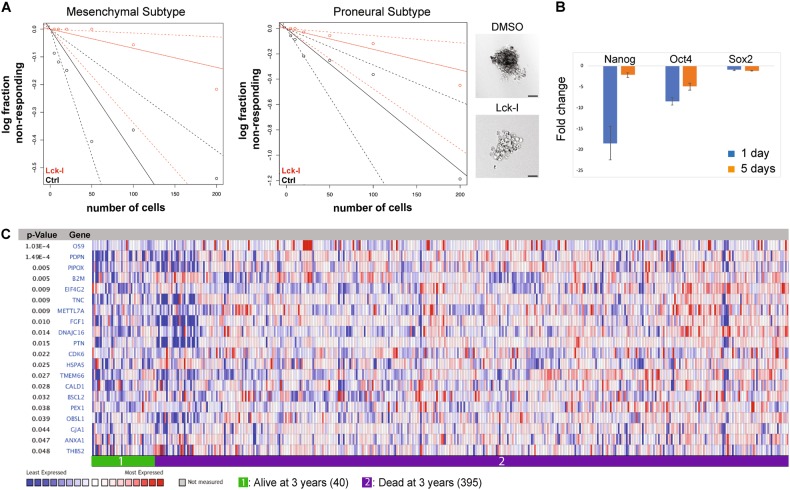
Lck-I attenuates self-renewal ability of hGSCs and inhibits Nanog-targeted genes that associate with decreased patient survival. **a** Limiting dilution analysis of patient-derived hGSCs with and without the addition of Lck-I, shows that Lck-I attenuates the self-renewal ability of hGSCs. The experiments were repeated six times and significance was calculated with a Chi-square test (*p* < 0.05). Photomicrographs show representative example of DMSO (control)-treated hGSCs that form floating tumor-spheres and Lck-I- treated hGSCs that cannot form tumor-spheres. Scale 100 μm. **b** qRT-PCR for the expression of stemness genes Nanog, Oct4 and Sox2 following treatment of hGCs with Lck-I for 5 days. Results are presented as fold change of expression compared to non-treated cells. Expression of Nanog and Oct4 are significantly inhibited after treatment with Lck-I (*n* = 3, Significance cutoff: two-fold). **c** Comparison of the Nanog-targeted genes inhibited by the Lck-I in vivo with the genes expressed in patients of the TCGA database that were diseased (395) or alive [38] 3 years after the initial diagnosis. The heat-map shows that 20 Nanog-targeted genes inhibited by the Lck-I treatment, belong to the top 10% of overexpressed genes in tumors of 395 diseased patients (*p* < 0.05)

To examine whether treatment with Lck-I affects the ability of hGSCs to self-renew, we cultured hGSCs from two patients with glioblastoma, in the presence of Lck-I for 7 days. This showed that Lck-I significantly decreased the tumor-sphere formation frequency of hGSCs as examined by in vitro extreme limiting dilution analysis (ELDA), a method widely used to determine self-renewal capacity [37] (Fig. 7a; *p* < 0.05). Since treatment of hGCs with Lck-I results in impaired self-renewal we hypothesized that Lck-I could affect expression of stemness-related transcripts. We performed qRT-PCR for the expression of Nanog, Oct4, and Sox2 in hGCs treated with Lck-I for 1 day or 5 days. We show that Lck-I significantly inhibits the expression of Nanog and Oct4 while Sox2 expression is inhibited but not significantly (Fig. 7b).

### Local treatment with Lck-I inhibits Nanog-target genes associated with glioblastoma patient survival

Since treatment with Lck-I results in significant inhibition of Nanog-targeted genes (Fig. 6e) and significant impairment of hGSC self-renewal (Fig. 7a), we sought to determine if the Nanog-targeted genes inhibited by the Lck-I might have a role in glioblastoma patient survival. We used the Oncomine™ Platform (Thermo Fisher, Ann Arbor, MI) for analysis and visualization of 435 glioblastoma patient samples in the TCGA database. We compared the Nanog-targeted genes inhibited by the Lck-I in vivo (Supplementary Table 1), with the genes expressed in patients that were diseased (395) or alive [38] 3 years after the initial diagnosis. Our analysis showed that 25% of the Nanog-targeted genes inhibited by the Lck-I treatment, belong to the top 10% of overexpressed genes in tumors of 395 diseased patients (Fig. 7c; *p* < 0.05). This suggests that local treatment using the Lck-I regulates the expression of clinically relevant target genes for human glioblastoma.

## Discussion

Glioblastomas are aggressive neoplasms due to the high rate of cancer cell migration and invasion into the surrounding brain parenchyma [39]. Current treatment of glioblastomas includes maximal surgical resection of the tumor mass followed by radiation to the region of the brain exhibiting abnormal signal on imaging plus a margin [33], the administration of temozolomide and electrical field antimitotic treatment [40]. Even with this multi-therapeutic approach, tumor recurrence is usual and the prognosis is not favorable. A portion of this is due to the migration properties of the tumor cells, which invade the brain parenchyma [38] that makes their elimination impossible with local or regional therapies.

It has been noted that glioma cells migrate preferentially in association with myelinated tracks [41] in the brain. Until now it was not possible to study the real-time interaction of hGCs with myelinated or non-myelinated tracks due to limitations in current models. Our ex vivo co-culture system resolves this issue by enabling live imaging of hGCs during their interaction with myelinated or non-myelinated axons. Although DRG axon cultures do not precisely represent the CNS environment and complexity, oligodendrocyte myelination of DRG axons is generally accepted as the best in vitro method to study CNS myelin and the interactions of CNS myelinated axons. Using these cultures, we demonstrate that glioma cells form extended pseudopodia to explore the surrounding microenvironment, interact with axons and migrate along axonal paths. Local exploration of the surrounding environment depends on the ability of the cell to interpret extracellular cues and adopt gene expression and protein synthesis in response to these signals. Positioning the relevant mRNA transcripts at the appropriate place within a cell enables an accelerated response to signaling inputs. With mRNAs concentrated at distinct locations, there is little time spent moving proteins through large regions of cytoplasm [42]. A well-described example is the response of the growth cone to attractive or repulsive cues, which is dictated by the local translation of specific mRNAs [43]. Such stimulus-driven mRNA-specific local translation spatiotemporally links signal reception to gene function [42] and is particularly relevant to the regulation of cancer cell migration and invasion. Here we show spatial localization of mRNAs for Lck, Paxillin, CrkII, and Rac1 in pseudopodia of hGCs. These transcripts associate with polyribosomes locally within the pseudopodia to undergo local translation. This suggests an important role in the dynamic regulation of cytoskeletal rearrangements of hGCs during exploration of their microenvironment and subsequently in control of their migration.

Several new therapies for glioblastomas, including EMD121974 (Cilengitide), target glioma cell migration and invasion [44]. A770041, which was developed as a targeted Lck inhibitor, targets a downstream effector of the integrin signaling pathway and thus can be more specific with respect to the cytoskeletal changes that control glioma cell migration than Cilengitide. Second, phospho-Lck is expressed preferentially in glioma cells, which makes it far more selective than integrins, which are expressed in endothelial cells as well. Although, A7700451 is highly potent inhibitor of Lck activity, it also binds at least 30 more kinases and receptors with high affinity [30], which may contribute to its biological potency against human glioblastoma and explain the extent of transcript inhibition that we observed in vivo. To determine potential side-effects of A770041 as local treatment for human glioblastomas, we have initiated pharmacokinetic and toxicology studies in rodents following intraventricular application of the inhibitor.

A prominent feature of glioblastoma is the presence of a distinct glioma stem cell population that is responsible for tumor propagation, growth, therapeutic resistance and recurrence. Several studies have shown the presence of a core embryonic stem cell-like stemness signature in glioblastomas consisting of *NANOG, OCT4*, and *SOX2* [45]. Inhibition of this stemness gene signature reduces glioblastoma growth and the number of GSCs. Nanog in particular, is required for the pluripotency of embryonic stem cells [46] and together with Sox2 and Oct4 they form a core stem cell network that control pluripotency and stemness, promoting stem cell expansion and self-renewal [47]. Lck mediates the expansion of the CD133+ GSC pool following ionizing radiation of glioblastomas and inhibition of Lck inhibits the radiation-induced expression of CD133, Nestin, and Musashi in GSCs [48]. The inhibition of Nanog target genes by Lck-I in our glioblastoma xenograft model, could explain the significant reduction in tumor size observed in treated animals. How Lck signaling integrates with known pathways that regulate Nanog target genes is currently unknown. The possibility that Lck could modulate the Hedgehog-Gli signaling network in glioblastomas as shown in T cells [49], to control glioma stemness and Nanog expression, is very intriguing and may open new possibilities for therapeutic interventions.

Glioblastoma is one of the most aggressive and fatal human tumors despite current therapeutic approaches. This makes the identification of molecular mechanisms that regulate glioma cell migration and stemness a critical endeavor. Here, we demonstrated that treatment of human glioblastomas with Lck-I results in significant inhibition of tumor growth, self-renewal of hGSCs, and expression of clinically relevant Nanog-targeted genes that associate with patient survival. Hence, we propose that local in vivo inhibition of Lck constitutes a promising new therapeutic approach for human glioblastomas.

### Data and materials availability

The RNAseq data from the Lck-I-treated tumors discussed in this publication have been deposited in NCBI’s Gene Expression Omnibus and are accessible through GEO Series accession number GSE95289:

## Materials and methods

### Isolation and culture of hGSCs

The institutional review board at Geisinger Clinic approved the collection of patient-derived glioblastoma multiforme (GBM) tissue. Primary hGSC spheres were cultured from human glioma samples as previously described [13]. The molecular background of the glioblastomas used in this study are included in Supplementary Table 3. All hGCs used in this study were authenticated by ATCC using short tandem repeat (STR) analysis. All human primary cells used were between passages 5 and 10. All cultures were routinely tested for mycoplasma contamination using the LookOut Mycoplasma PCR Detection kit (Sigma).

### Oligodendrocyte-DRG neuron co-cultures

The cortices from P2 rat pups were dissected and diced with a scalpel followed by dissociation by papain (Worthington) and DNase I (Sigma) at 37 °C for 80 min. Papain buffer was removed and tissue triturated in media containing 10%FBS (Life Technologies) three times, until completely dissociated. Cells were pelleted and resuspended in DMEM including 0.5% BSA and ITS (Life Technologies), filtered through a 30 μm mesh filter, then incubated at 37 °C for 15 min on a non-cell-culture treated 100 mm dish to allow microglia attachment. Floating cells were collected, centrifuged, and anti-A2B5-magnetic bead labeling was conducted according to manufacturer’s protocol (Miltenyi Biotec). Purified cells were resuspended in N2B2 media and seeded on rat DRG neurons isolated as previously described [16, 50], and maintained in N2B2 + T3 (R&D Systems) media for 10–14 days to allow for myelination.

### Immunocytochemistry

The following primary antibodies were applied overnight at 4 °C: Nestin and NeuN (Millipore), GFAP (DAKO), A2B5 (R&D Systems), Mushashi1, Nanog, and Sox2 (Cell Signaling). Slides were examined using a Zeiss Axiovert fluorescent microscope.

### Quantification of pseudopodia formation

Cells were seeded on fibronectin-coated (10 μg/ml, Sigma) glass chamber slides (NUNC) in the presence of 10% FBS for 2.5 h. Cultures were treated with 500 nM of Lck inhibitor (A770041, Axon Medchem) or DMSO vehicle for 2 h and then fixed in 4% formaldehyde. The samples were stained overnight at 4 °C with Phospho-Paxillin antibody (CellSignaling). The following day, secondary antibodies were applied followed by application of Rhodamine RedX Phalloidin (Life Technologies) and Hoechst stain (Life Technologies). Slides were imaged using a Zeiss LSM 750 confocal microscope.

### Immunohistochemistry

Tissue microarrays containing paraffin-embedded samples of normal brain, GBM, astrocytoma, oligodendroglioma, and meningioma tissues were obtained from US Biomax, Inc. Tissue sections were incubated overnight with phospho-Lck (Y394) and phospho-Lck (S158) at a concentration of 10 μg/ml (our own pLck-Y394 antibody [25] and Abcam, respectively). Images were captured with a Zeiss Axiovert inverted microscope at ×20 magnification.

### Immunoprecipitation and western blotting

Primary antibodies against phosphorylated and total Lck, Src, Fyn, Lyn, or Yes (Cell Signaling) and the lysates were incubated overnight at 4 °C with gentle rotation. The following primary antibodies were used: p(Y418)-Src family, Lck (D88), p(Y188)-paxillin, pCrkII, Histone H3, Lamin A/C (Cell Signaling), and β-actin (Sigma). Densitometric analysis was conducted with FluorChem SP analytical software.

### xCELLigence

Electrical impedance due to cell migration is represented as cell index. hGCs were seeded on fibronectin-coated wells of the RTCA CIM-plate 16 (ACEA Biosciences) and allowed to attach and spread for 1 h at room temperature. After 1 h, wells were treated with DMSO vehicle or 500 nM Lck-I. As cells migrated towards the lower chamber containing 10% FBS with DMSO vehicle or 500 nM Lck-I, measurements were captured every 5 min. Cell index data was plotted overtime and the slope between 1 and 10 h was calculated.

### Cell motility qPCR array using RNA from hGC pseudopodia

RNA transcripts within the hGC pseudopodia were determined via qRT-PCR. Total RNA was isolated from pseudopodia that had migrated through 1 μM pores of 10 μg/ml human fibronectin-coated six-well inserts (BD Falcon) for 24 h. 900 ng of total RNA from each hGC pseudopodia sample was reverse-transcribed using the RT^2^ First Strand Kit (Qiagen). Quantitative PCR was performed using the Human Cell Motility RT^2^ PCR Array (Qiagen). The relative abundance of each transcript (*C*_t_ < 35) is represented by a gene heat map from *GAPDH* normalized *C*_t_ values using GENE-E software (Broad Institute).

### Polysome fractionation and RNA isolation

Polysome-associated RNA from hGSc was isolated as described by Gandin et al. [26]. Briefly, following sucrose gradient centrifugation, polysomes were fractionated using a gradient station (BioComp) for continuous fractionation. Polysome-associated RNA was then isolated from the polysome containing fragments using standard RNA extraction protocol. Approximately 100 ng of total RNA was reverse-transcribed using the RT^2^ PreAMP cDNA Synthesis Kit (Qiagen) followed by qPCR for *Lck, CrkII, Paxillin, and Rac1* (Qiagen). The expression level of each RNA transcript (*C*_T_ < 40) was normalized to *GAPDH*.

### Stereotactic injections

To determine the number of animals (*n* = 10 animals per group) we performed a power analysis assuming results with confidence level above 90%. Intracranial injections were performed using a stereotaxic apparatus (Kopf) on 8-week-old Nu/J male mice (Jackson Laboratories) initially sedated with 4% isoflurane and maintained with 2% isoflurane. After leveling the skull, a hole was drilled with a #72 micro drill bit (Kyocera) at coordinates −2.0 mm AP and +1.5 mm ML relative to Bregma. A 75 RN Hamilton syringe was then lowered to a depth of −2.5 mm DV at a rate of .5 mm per minute, and 200,000 primary hGSCs resuspended in a total volume of 4 μl were injected at a rate of 0.5 μl per minute using a Stoelting Quintessiential Stereotaxic Injector. To reduce backflow, the syringe rested for 2 min post-injection, before it was withdrawn at a rate of 0.5 mm per minute, and the cavity was immediately sealed with bonewax (Ethicon). A subcutaneous pocket was created, and an Alzet osmotic pump 1004 fitted with brain infusion kit 3 and either 1.175 mg of Lck-I (Axon Medchem) or DMSO/Kolliphor ELP vehicle. A second hole was drilled at coordinates of +0.5 mm and +1.1 mm ML relative to Bregma with the same micro-drill bit to place the brain infusion catheter in the right lateral ventricle. The catheter was lowered into the ventricle and glued to the skull using Loctite 454 cyanoacrylate adhesive. After 4 weeks, brains were harvested, post-fixed in 4% formaldehyde, paraffin embedded, sliced, and stained with HuNu antibody (Abcam). Since the ALZET pumps can deliver the Lck inhibitor for a maximum of 4 weeks, we could not perform meaningful survival studies. It is theoretically possible to exchange or refill the pumps but when we tried this, we noticed various technical issues with the intracranial catheters that had adverse effects on the survival and overall health of the mice. Hence, we decided not to pursue survival studies

### 3D brain reconstruction and analysis

HuNu-stained brain slices from the Nu/J mice used in the stereotactic experiments were imaged at 10× (Zeiss) and stacked. Each mouse brain was represented by 30 consecutive 40 µM-thick slices at an interval of 120 µM between slices and were stacked and aligned using Brainmaker software (MBF Biosciences). HuNu-stained contours within this 3D stack were then manually traced for each slice, and a 3D reconstruction of each tumor was created using Neurolucida (MBF Biosciences). Quantification of each tumor was achieved using Neurolucida Explorer (MBF Biosciences). Statistical significance was determined by the two-tailed Student’s *t*-test.

### RNA-seq and data analysis

RNA-seq was performed using Illumina HiSeq2500 (GeneWiz). The 50-nucleotide sequence reads were aligned to the hg19 build of the human genome using *gsnap* [51]. The genomic locations of genes and exons defined in Refseq were extracted from the *refGene.txt* (http://hgdownload.cse.ucsc.edu/goldenPath/hg19/database/refGene.txt.gz). Read summarization at the gene level was done with in-house scripts using reads with a mapping quality of 20 or greater. Gene expression was analyzed in R with the *limma* package [52, 53], applying voom precision weights to account for the mean-variance dependency observed in the standardized read counts [54]. Differential gene expression analysis was done by applying the decideTests routine to the eBayes fit of the contrast of the samples treated or not treated with the Lck-I, using the Benjamini–Hochberg method to control the false discovery rate [55]. Genes with an adjusted *p*-value < 0.05 were collected for functional analysis, done using the Gene Set Enrichment Analysis [56] at http://software.broadinstitute.org/gsea/index.jsp

### Analysis of TCGA datasets

Data for the figures were obtained from a dataset of RNA sequencing of 667 glioblastoma and low-grade glioma samples in The Cancer Genome Atlas hosted on the GlioVis data visualization portal. Figures were plotted using R version 3.4.4 (R Foundation for Statistical Computing, Vienna, Austria)

### qRT-PCR for stemness transcript expression

100,000 primary glioma stem cells per well were seeded in a non-treated 24-well plate and grown in suspension over the first 24 h without any treatment. Subsequently, neurospheres were treated with a final A77 concentration of 500 nM or the equivalent volume of DMSO once per day for 5 days. Total RNA was isolated at day 1 and day 5 post-treatment using the Arcturus PicoPure RNA Isolation Kit (ThermoFisher), and RT-qPCR was performed on 700 ng of each RNA sample using the RT^2^ First Strand Kit and RT^2^ Primer Assays (Qiagen) for stemness markers NANOG, OCT4, and SOX2.

### Statistical analysis

Our goal is to obtain results with greater than 90% confidence level. We used the D’Agostino and Pearson normality test and the Shapiro–Wilk normality test to determine the distribution of our individual data sets, which determined that our data follow a Gaussian distribution. The homogeneity of variances was confirmed with Brown and Forsythe test and assuming that the standard deviation for measurements is no more than 3/4 of the mean, a two-tailed Student’s *t*-test was employed to compare between two sets. Significance was determined when *p* < 0.05. For determination of Stemness using StemChecker, the significance was calculated by the hypergeometric test, which assess the enrichment of stemness signature genes against the full annotated human genome. The adjusted *p*-value was calculated by the Bonferroni correction.

## Electronic supplementary material

Supplemental Figure legends

Supplemental Table 1

Supplemental Table 2

Supplemental Table 3

Supplemental Figure 1

Supplemental Figure 2

Supplemental Figure 3

Supplemental Figure 4

Supplemental Figure 5

Movie S1

Movie S2

Movie S3
