# Supplementary material for: Regulation of human glioma cell migration, tumor growth, and stemness gene expression using a Lck targeted inhibitor
Source: Oncogene. 2018 Oct 23;38(10):1734–50. doi: 10.1038/s41388-018-0546-z (PMC6462869; doi:10.1038/s41388-018-0546-z)
Supplement: Supplementary file 1 — Supplemental Figure legends [file 41388_2018_546_MOESM1_ESM.docx]

**Figure S1: Isolated human glioma stem cells express the stem cell markers Nestin, CD133, Mushashi1, Nanog and Sox2 and retain the capacity to differentiate in vitro and in vivo.** A) Glioma stem cells (GSCs) isolated from patients with primary GBM and cultured in Neurobasal-A media supplemented with bFGF, EGF and heparin express the stem cell markers Nestin, CD133, Mushashi1, Nanog and Sox2. B) Addition of 10%FBS and removal of bFGF, EGF and heparin for 7 days induces differentiation of GSCs into GFAP+ astrocytes, A2B5+ oligodendrocytes and NeuN+ cells. C) Orthotopic xenograft transplantation of the hGSCs into nude mice gives rise to human glioblastomas as seen by H&E stain. Tumors were stained positive for the proliferative marker Ki67 and expressed the astrocytic marker GFAP and the oligodendrocyte-specific marker Olig2.

**Figure S2:** A) Heat map of RNA-seq data from three patient-derived hGSCs and two HNSCs shows elevated expression of Lck, Paxillin, CrkII and Rac1 (red arrows and rectangles) in HGSCs. Yellow color corresponds to high expression and blue to low expression. B) Comparison of the Lck transcript expression levels between control (healthy) brain and glioblastoma tissue samples using the TCGA database. The expression of Lck is significantly higher in glioblastomas (p<0.03; Welch t-test, 95% CI:

-0.3682 to -0.0132). C) Staining of a human GBM tissue array containing 40 GBMs with an antibody against phospho-Lck (S158) shows that 90% of GBMs express phosphorylated Lck. D) Treatment of hGSCs with 500nm Lck-I does not affect the phosphorylation state of Src, Fyn, Lyn and Yes suggesting that the Lck-I is highly specific.

**Figure S3:** A) Wound healing “scratch” assay using cultures of confluent human glioma cells. 32 hours post injury the wound is not healed in the presence of Lck-I as compared to control non-treated cells. The area of the wound was measured at T0 (immediately after the wound) at T24 (24 hours later) and at T32 (32 hours later) using ImageJ64 software and plotted as percentage of the total area at 10X magnification. The results are the average of 5 independent experiments and show that 24 and 32 hours after the wound the control cells migrate and cover a significant portion of the wound area (p<0.05), while the Lck inhibitor-treated cells do not migrate efficiently. B) hGSCs were seeded on 3D Alvetex scaffolds (Reinnervate) and left untreated or treated with Lck-I for 72 hours. Confocal z-stack images show that inhibition of Lck results in significant inhibition of the invasion properties of human glioma cells. Quantification of the depth of invasion was performed from 3 independent experiments and significance was calculated with two-tailed student’s t-test (*:p<0.05). C) In vitro cytotoxicity and proliferation assays in untreated hGSCs or treated with Lck-I for 1 day and 5 days. Lck-I does not affect the viability or the proliferation of hGSCs.

**Figure S4:** Representative hematoxylin & eosin staining of brain tissue slides from one control mouse and two Lck-I treated mice 4 weeks after intracranial implantation of 200,000 patient-derived human glioma cells. Arrows indicate the gross boundaries of the tumors. Lck-I treated mice show minimal invasion of human glioma cells into the brain parenchyma.

**Figure S5:** qPCR expression analysis of the Lck-I inhibited/Nanog-targeted genes that belong to the 10% of overexpressed genes in human glioblastomas according to the TCGA, 1 day and 5 days after in vitro treatment of hGSCs with Lck-I. Lck-I downregulates the expression of most of these transcripts in hGSCs. The experiment was repeated three times and the results are presented as average +/- St.Dev. Significance was calculated using an unpaired Student’s t-test (n=3, *: p<0.05, df=2).

**Movie S1: GFP+ hGSCs interact with DRG axons through the formation of pseudopodia:** Movie showing extensive interaction of hGSCs with axons and “pulling” of individual axon fibers. The movie was composed of serial images taken every 10 minutes over the course of 5 days.

**Movies S2 & S3: 3D reconstruction of brain slices in Lck-I and control treated animals with orthotopic human glioblastoma xenografts.** Inhibition of Lck results in significant reduction of tumor growth. Red contours depict the outline of the brain and green contours outline the tumor.
