# Supplementary material for: Regulation of human glioma cell migration, tumor growth, and stemness gene expression using a Lck targeted inhibitor
Source: Oncogene. 2018 Oct 23;38(10):1734–50. doi: 10.1038/s41388-018-0546-z (PMC6462869; doi:10.1038/s41388-018-0546-z)
Supplement: Supplementary file 2 — Supplemental Table 1 [file 41388_2018_546_MOESM2_ESM.pdf]

Nanog-targeted genes inhibited by Lck-I in vivo

BMP7
DPYSL3
FGF2
GJA1
TNC
THBS2
ZEB2
OBSL1
ICMT
PIPOX
C12orf35
H2AFJ
LRRN1
FAM33A
ACADM
ADAR
ANXA1
ASAH1
B2M
CALD1
CAV1
CDK6
CFL1
VCAN
DCX
EIF4G2
FAT
FDPS
GOLGA4
H2AFZ
HSPA5
CYR61
PEX1
PRCP
PSMB1
PTN
PTPN1
RBM4
RPLP1
SEPP1
SGK

ZNF217
ZNF226
PICALM
NOLC1
EPM2AIP1
ACOT8
PDPN
SERINC3
WDR6
DNAJC16
METTL7A
POLR1A
CLIC4
BSCL2
GOLIM4
HSD17B12
LARP7
TMEM66
CYB5B
DCTN5
HIST3H2A
TMEM123
ZNF664
ZNRF2
KLF15
NFIX
ACSBG1
EPHA7
FGF1
HIST1H1E
SLC15A2
ZNF224
HIST1H4D
CALCRL
OS9
ACCN4
LMO3
GRAMD3
ZNF436
CD99L2
ZNF484
C6orf204
