# Supplementary material for: Regulation of human glioma cell migration, tumor growth, and stemness gene expression using a Lck targeted inhibitor
Source: Oncogene. 2018 Oct 23;38(10):1734–50. doi: 10.1038/s41388-018-0546-z (PMC6462869; doi:10.1038/s41388-018-0546-z)
Supplement: Supplementary file 3 — Supplemental Table 2 [file 41388_2018_546_MOESM3_ESM.pdf]

Genes that appear in both

Primary Concept:	Nanog target genes
Associated Concept:	Glioblastoma vs. Normal - Top 10% Over-expressed (TCGA Brain)
Interaction	P-value: 1.10E-6 Q-value: 0.003 Odds Ratio: 4.0 Size: 22 genes
Symbol	Gene Name
ACADM	acyl-CoA dehydrogenase, C-4 to C-12 straight chain
ANXA1	annexin A1
ASAH1	N-acylsphingosine amidohydrolase (acid ceramidase) 1
B2M	beta-2-microglobulin
CLIC4	chloride intracellular channel 4
EIF4G2	eukaryotic translation initiation factor 4 gamma, 2
FGF1	fibroblast growth factor 1 (acidic)
FGF2	fibroblast growth factor 2 (basic)
GJA1	gap junction protein, alpha 1, 43kDa
H2AFZ	H2A histone family, member Z
LARP7	La ribonucleoprotein domain family, member 7
METTL7A	methyltransferase like 7A
PICALM	phosphatidylinositol binding clathrin assembly protein
PRCP	prolylcarboxypeptidase (angiotensinase C)
PTN	pleiotrophin
SEPP1	selenoprotein P, plasma, 1
THBS2	thrombospondin 2
TMEM123	transmembrane protein 123
TNC	tenascin C
VCAN	versican
ZEB2	zinc finger E-box binding homeobox 2
ZNF217	zinc finger protein 217
