# Supplementary material for: Regulation of human glioma cell migration, tumor growth, and stemness gene expression using a Lck targeted inhibitor
Source: Oncogene. 2018 Oct 23;38(10):1734–50. doi: 10.1038/s41388-018-0546-z (PMC6462869; doi:10.1038/s41388-018-0546-z)
Supplement: Supplementary file 4 — Supplemental Table 3 [file 41388_2018_546_MOESM4_ESM.pdf]

	Cosmic ID	Nucleotide mutation	Amino acid substitution

Neural

none

Proneural

	Cosmic ID	Nucleotide mutation	Amino acid substitution
IDH1	28748	c.394C>A	p.R132S
IDH1	28747	c.394C>T	p.R132C
IDH2	34039	c.514A>T	p.R172W
NRAS	564	c.35G>A	p.G12D

Mesenchymal

	Cosmic ID	Nucleotide mutation	Amino acid substitution
EGFR	21687	c.866C>T	p.A289V
IDH1	28748	c.394C>A	p.R132S
IDH1	28747	c.394C>T	p.R132C
NF2	23667	c.1396C>T	p.R466*
NRAS	564	c.35G>A	p.G12D
