# Supplementary figures and images for: Regulation of human glioma cell migration, tumor growth, and stemness gene expression using a Lck targeted inhibitor
Source: Oncogene. 2018 Oct 23;38(10):1734–50. doi: 10.1038/s41388-018-0546-z (PMC6462869; doi:10.1038/s41388-018-0546-z)

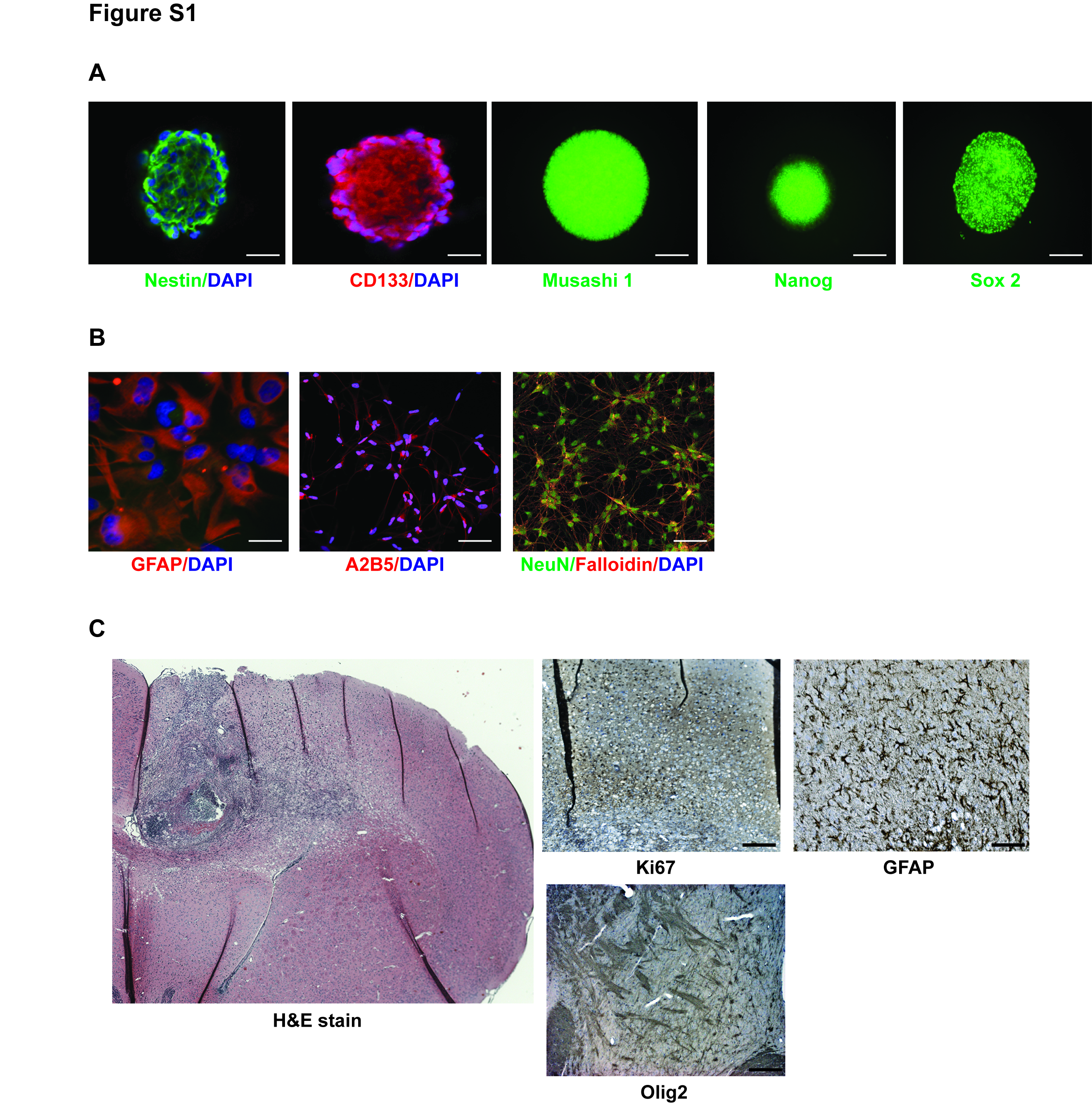

Supplement: Supplementary file 5 — Supplemental Figure 1 [file 41388_2018_546_MOESM5_ESM.tif]

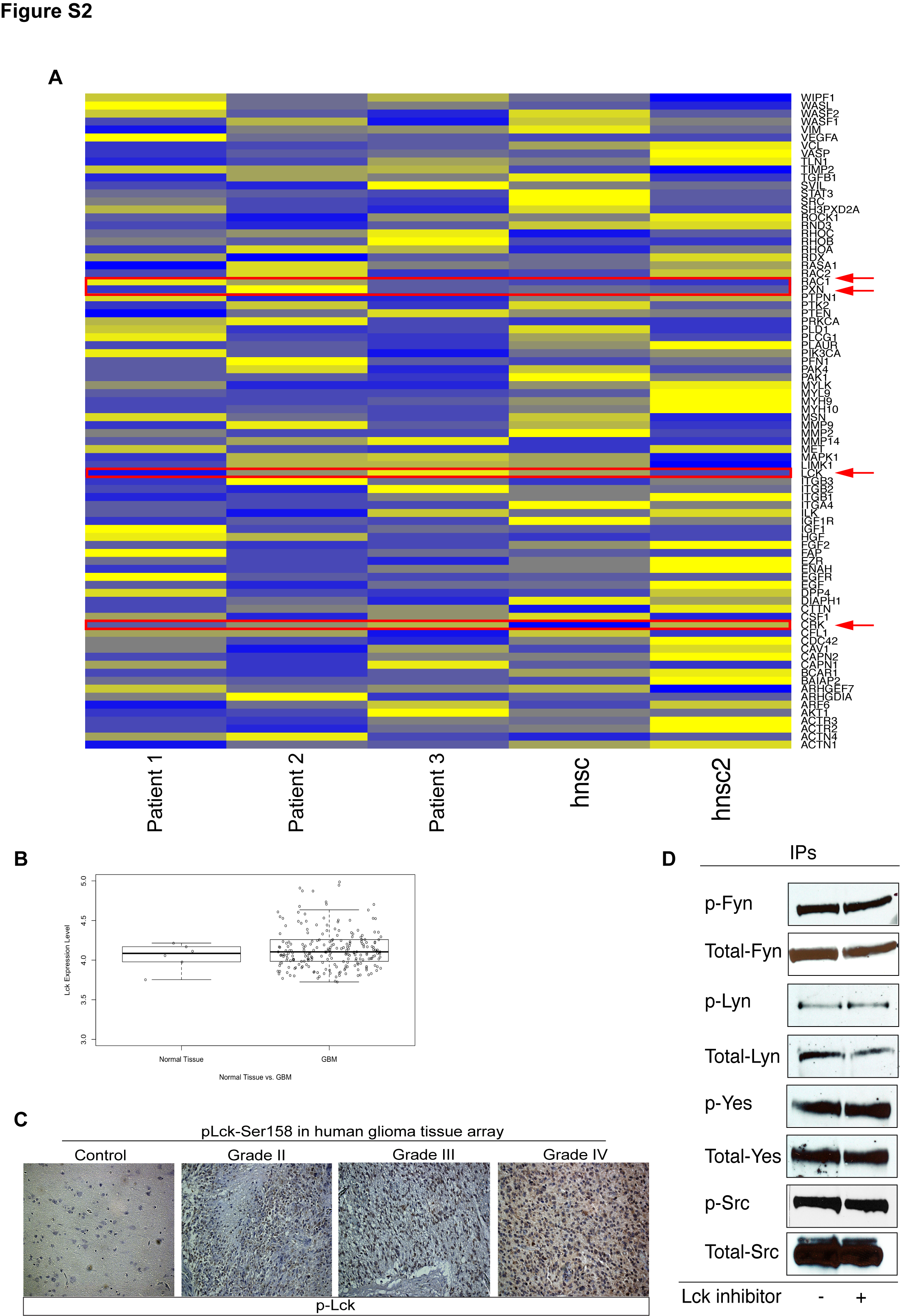

Supplement: Supplementary file 6 — Supplemental Figure 2 [file 41388_2018_546_MOESM6_ESM.tif]

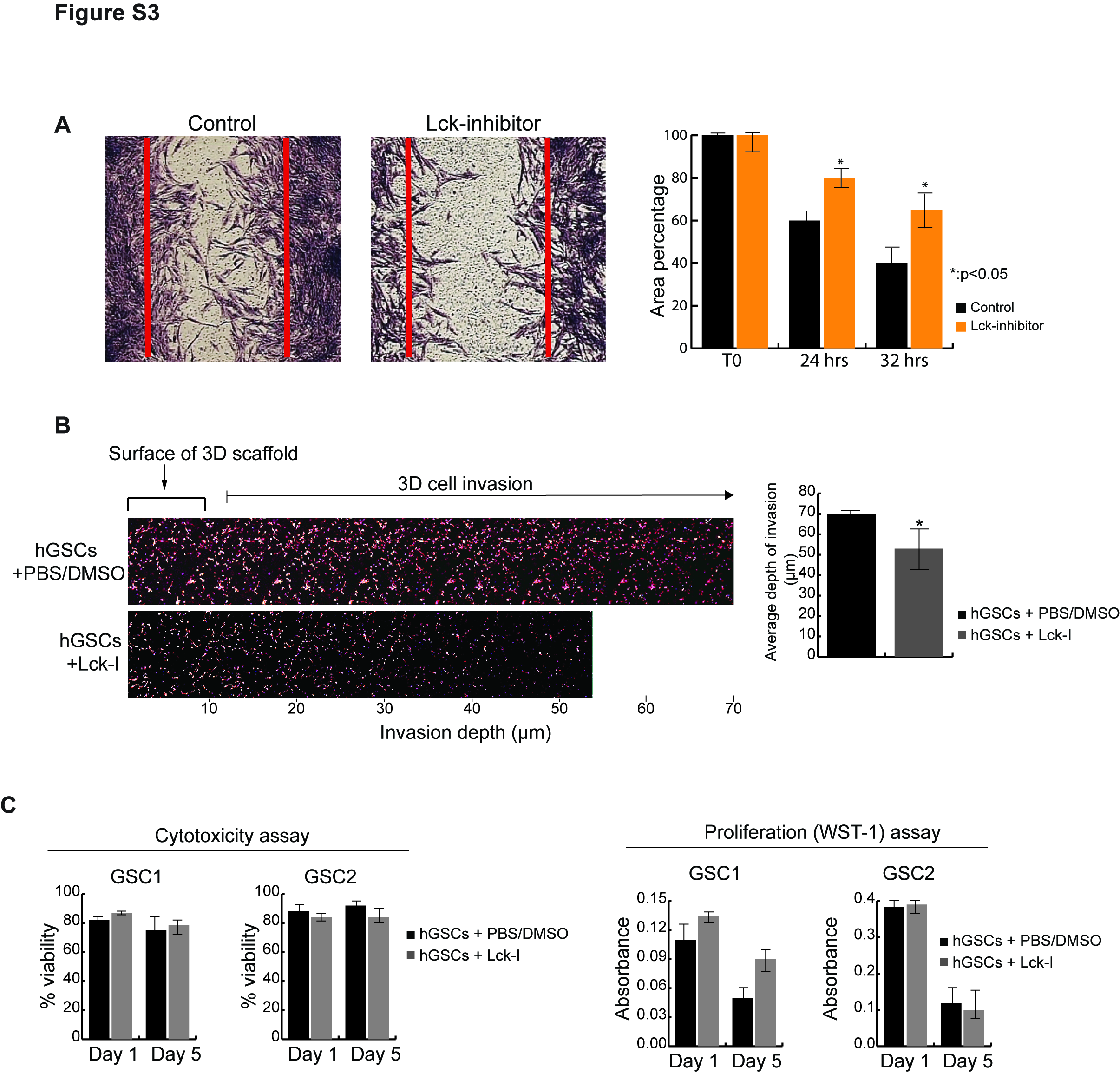

Supplement: Supplementary file 7 — Supplemental Figure 3 [file 41388_2018_546_MOESM7_ESM.tif]

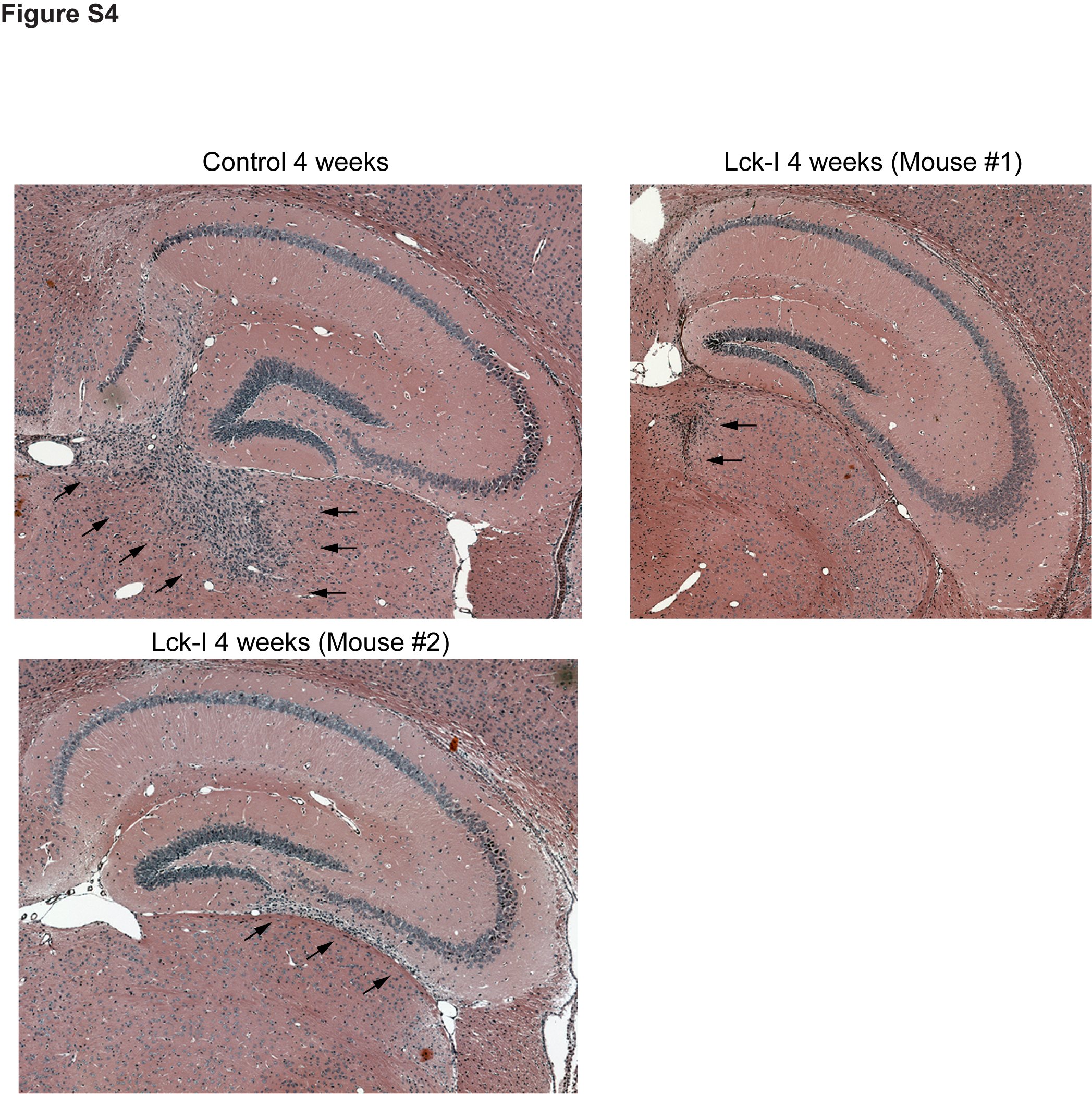

Supplement: Supplementary file 8 — Supplemental Figure 4 [file 41388_2018_546_MOESM8_ESM.tif]

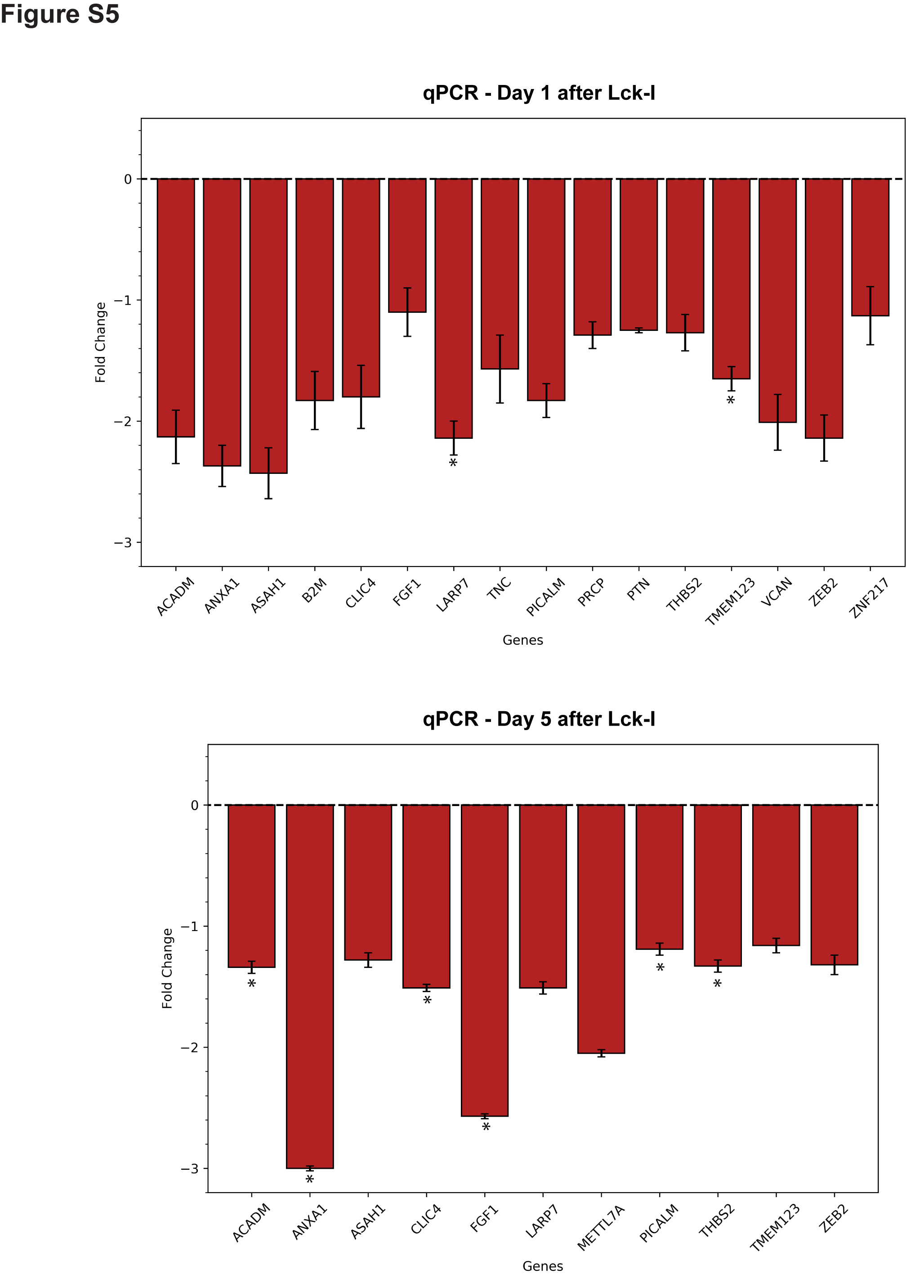

Supplement: Supplementary file 9 — Supplemental Figure 5 [file 41388_2018_546_MOESM9_ESM.tif]
